# Translocation of Loline Alkaloids in *Epichloë*-Infected Cereal and Pasture Grasses: What the Insects Tell Us

**DOI:** 10.3390/jof9010096

**Published:** 2023-01-10

**Authors:** Alison J. Popay, Joanne G. Jensen, Wayne R. Simpson, Wade J. Mace, Chanatda Somchit

**Affiliations:** 1 AgResearch Ltd., Ruakura Research Centre, Private Bag 3123, Hamilton 3240, New Zealand; 2 AgResearch Ltd., Grasslands Research Centre, Tennent Drive, Palmerston North 4410, New Zealand

**Keywords:** phloem, xylem, rye, *Elymus*, ryegrass, tall fescue, meadow fescue, aphids, spittlebug, endophyte

## Abstract

Aphids are major pests of cereal and pasture grasses throughout the world, vectoring disease and reducing plant production. There are few control options other than insecticides. *Epichloë* endophytes that produce loline alkaloids in their hosts provide a possible mechanism of control, with both meadow fescue and tall fescue naturally infected with loline-producing endophytes showing a resistance to *Rhopalosiphum padi.* We screened *Elymus* spp. naturally infected with endophytes that produced loline alkaloids at concentrations known to affect aphids on fescue but found no effect on these insects infesting *Elymus*. A synthetic loline-producing endophyte association with rye also had no effect on the aphids. After hypothesizing that the lolines were being translocated in the xylem in *Elymus* and rye rather than the phloem, we tested the rye and meadow fescue infected with loline-producing endophytes against a xylem feeding spittlebug. The endophyte in rye inhibited the feeding of the insect and reduced its survival, whereas the endophyte-infected meadow fescue had no effect on the spittlebug but reduced the number of aphids. Lolines applied to the potting medium of endophyte-free and endophyte-infected rye, ryegrass, and tall fescue resulted in a decrease in the aphid populations on the endophyte-free pasture grasses relative to the untreated controls but had no effect on aphid numbers on the rye. We tentatively conclude that lolines, produced in both natural and synthetic association with *Elymus* and rye, are partitioned in the xylem rather than the phloem, where they are inaccessible to aphids.

## 1. Introduction

Insects that specialize in sap feeding cause extensive losses in agriculture around the world. Most tap into the living cells in the nutrient-rich phloem, sucking up resources that would otherwise be used by the plant for its growth and reproduction. Phloem feeders such as *Rhopalosiphum padi* and *Sitobion avenae* are notorious for vectoring diseases such as the cereal yellow dwarf diseases which reduce photosynthesis, cause chlorosis, and stunt plant growth [1,2]. Worldwide, yield losses to the Barley Yellow Dwarf Virus (BYDV), the most common of these diseases, are estimated to be as high as 80% [1]. *Diuraphis noxia*, or the Russian wheat aphid, which has in recent years extended its range to include many of the world’s cereal-growing areas, causes leaf rolling, chlorotic streaking, and stunting, resulting in substantial yield losses, but it is a poor transmitter of the virus [3]. Other sap-feeders feed on the more dilute xylem and some insects can exploit both the phloem and the xylem [4,5]. Other diseases such as *Xylella fastidiosa*, a particularly virulent bacterium threatening olive groves and grapevines, are spread by xylem feeders such as leafhoppers and spittlebugs. Insecticides such as neonicotinoids have been the main control agent of these vectors in recent years, but concerns about the environmental consequences of their ongoing use have resulted in them being banned in Europe. Plant resistance to the diseases themselves has not so far been successful, while developing resistance to the vectors is also challenging [1].

As a source of food, members of the grass family Poaceae, including cereal and pasture grasses, are economically the most important plants in the world. Some of these host species of fungi in the genus *Epichloë,* forming mutualistic relationships with grasses of the sub-family Pooideae in which the endophyte relies on the plant for protection, transmission to the next generation and the resources for their growth and the production of alkaloids, which the plant benefits from as these alkaloids protect it from herbivory and provide resilience to abiotic stress.

Loline alkaloids are potent drivers of the anti-insect activity of *Epichloë* spp. that infect grasses such as tall fescue (*Festuca arundinacea*), meadow fescue (*Festuca pratensis*), and annual ryegrass (*Lolium multiflorum*) [6]. They are saturated amino pyrrolizidine alkaloids, unlike the hepatotoxic and carcinogenic unsaturated pyrrolizidine alkaloids that are also associated with plants [7]. They reduce the feeding of both sap sucking and chewing insects and have the advantage that they are translocated away from the mycelium, where they are manufactured, into the leaves and roots, affecting the invertebrates that feed on these parts of the plant.

Aphids, including the economically important *Rhopalosiphum padi*, are adversely affected by some of these alkaloids; in particular, by lolines [8,9,10,11]. Following the discovery that wild cereal relatives such as *Elymus* and *Hordeum* naturally host *Epichloë* spp. [12] provided some optimism that a source of aphid resistance would be found among these associations. More importantly, some of those strains were subsequently found to produce loline alkaloids [12].

The genesis of the research described in this paper was the finding that *Elymus* species hosting *Epichloë* endophytes that produce loline alkaloids did not provide the expected resistance to aphids. The result was the same when these endophytes were successfully inoculated into rye (*Secale cereale*), an *Elymus* relative. This led to the hypothesis that lolines are not being transported in the phloem of these species but instead are transported in the xylem. Further experiments were then carried out with xylem feeders, specifically spittlebugs (*Philaenius spumarius*), and a final experiment explored the effects of lolines applied to the roots on aphids infesting ryegrass, tall fescue, and rye.

## 2. Materials and Methods

### 2.1. Loline-Producing Epichloë Endophytes in Wild Elymus spp. and Rye (Secale Cereale)

Wild grass experiment: The seed of three species (*E. mutabilis, E. caninus,* and *E. uralensis*) was germinated in Petri dishes in mid-winter (July–August 2015) and then planted into a potting mix in individual pots from early August. They were maintained with regular watering in a glasshouse at an average temperature of 20 °C (range 18–25 °C). In late September, all plants with more than three tillers were tested for *Epichloë* using an immunoblot technique [13]. Plants where the blot results were equivocal, or that had been too small at the first blot dates, were tested on 27 October.

The plants of the three test species infected with loline-producing endophytes were part of a wider group of *Elymus* species that were tested in two groups in this trial. All loline-producing endophytes were in Group I. The endophyte infection levels varied across the group, resulting in a very low number of negative controls in some associations (Table 1). Both positive and negative plants were re-potted into a potting mix in 10.5 cm diameter (0.51 L) plastic pots in spring (12 October 2015). On 21 October, any aphids or aphid mummies (parasitoid-infected aphids) present on each plant were squashed or removed and the plant was trimmed to a 12 cm height. Each pot was placed inside a larger (15 cm diameter) pot, and five *R. padi* aphids were released onto each plant contained within a nylon mesh cage. The cages were removed after 1 week and the number of *R. padi* aphids on each plant was counted. Another aphid species, *Metopolophium dirhodum*, was also present and these were counted, but the data pertaining to them have not been presented here. There was no evidence, however, that this species was affected by endophyte.

The plant material was harvested, then freeze dried and finely ground. The loline alkaloids were measured using a modification of the gas chromatographic methods reported [14]. A sample of lyophilized grass tissue (50 mg) was extracted for 1 h with 50 µL of 40% methanol/5% ammonia and 1 mL of 1,2-dichloroethane (containing 53.7 ng of mL^−1^ 4–phenylmorpholine as the internal standard) followed by centrifugation at 5000× *g* for 5 min. The supernatant was transferred to amber glass GC vials via a 10 µm filter for the analysis. The analysis was conducted on a chromatography-flame ionization detector (Shimadzu GC17a; Shimadzu Corporation, Kyoto, Japan) equipped with a ZB-5 capillary column (30 m × 0.32 mm × 0.25 µm film; Phenomenex, Torrance, CA, USA). The limit of quantitation using this technique was 25 µg g^−1^.

For the statistical analysis, the total number of aphids and the total number of *R. padi* were analyzed using linear mixed effects models, fitted using GenStat V17. The data were natural log transformed prior to the analysis to stabilize the variance. Data from the two trial groups were analyzed jointly, although all the loline-producing endophytes were in the same group. The random effects of the group, and the row and column within group, accounted for the trial effects and spatial effects within the trials, respectively. The statistical significance of the treatment terms (the accession, endophyte status (E− vs. E+), and the accession by the endophyte status interaction) were assessed using approximate F statistics.

Rye Experiment I: Rye (cv Rahu) seedlings that had been inoculated with the loline-producing-endophyte AR3046 16 days previously and then grown on agar plates were transferred from AgResearch Grasslands to AgResearch Ruakura where they were to be investigated for their effect on nematodes. The plants were transplanted into a potting mix and initially kept in a controlled environment room before being transferred to a glasshouse. On 14 January, the immunoblotting showed, of 24 inoculated plants, that 12 were infected with endophyte and 12 were uninfected and therefore could be used as inoculated negative controls. A further five plants that had not been inoculated with endophyte were included as the uninoculated controls. On 30 January, the plants were transplanted into root trainers and subsequently developed a natural infestation of aphids. These aphids were suctioned from the plants on 13 February and preserved in 70% ethanol for later identification and counting. The number of tillers per plant was recorded.

Rye Experiment II: The effects of eight different *Epichloë* endophyte strains, all of which produced loline alkaloids, were investigated in mature rye plants. These plants were initially grown at AgResearch Grasslands before being transferred to AgResearch Ruakura in January 2014. Ten endophyte-free plants of the same age were included as the controls. The plants were cloned, and each clone was repotted into a sieved potting mix in 12 cm pots and placed in a glasshouse. One plant of each cloned pair was shifted to a controlled environment room set at 18 °C on 22 January. To provide additional replicates of the endophyte strain AR3050 in rye cv Rahu, three plants infected with AR3050 and three E− plants that had been grown from seed in 2013 were included in the experiment. The dead leaf sheath material and tillers were removed from all plants.

The aphids were taken from the plants on 20 January, kept in specimen vials in the 18 °C CE room, and were provided with wheat leaf segments in order to exclude the parasitized individuals. Three mature *R. padi* nymphs were placed on each plant and the remaining aphids were scattered over a tray of endophyte-free rye plants placed next to the experimental plants to act as a further source of aphids. The plants were not covered and were bottom watered as required. The aphids were suctioned from the plants between 11 and 18 February 2014 and then counted.

### 2.2. Comparative Effects on Aphids and Spittlebug

Two experiments were carried out with *R. padi* aphids and one with spittlebug. The experiments used 1-year-old rye plants cv. Rahu, either infected with the loline-producing endophyte strain AR3046 or endophyte-free. Meadow fescue plants with the loline-producing endophyte AR1006 [15] or endophyte-free were similarly potted up, allowing for a direct comparison of the effects of these loline-producing endophytes on aphids and spittlebugs in the different hosts. All experiments were comprised of six replicate plants for each treatment set up in randomized replicate rows.

For the spittlebug experiment in November 2013, six plants of each treatment, planted in a commercial potting mix in 7 cm diam. pots, were each placed into larger 12 cm diam. pots that were then enclosed in a mesh bag. The spittlebugs were collected mainly from Yorkshire fog (*Holcus lanatus*) on a farm close to Hamilton, New Zealand. Three nymphs were released onto each plant. The number of spittlebug adults and nymphs was recorded 24 h after the experiment was set up and again 5 days later. Assessments were made of the presence of spittle foam on the first and last 2 days, with each globule being categorized as small, medium, or large. To obtain a total score for all assessments, small, medium, and large globules of spittle were multiplied, respectively, by 0.5, 0.75, and 1. A final assessment was carried out on day 5.

The two aphid experiments were carried out between mid-November 2013 and January 2014 using the same plant treatments as for spittlebug. For the first experiment, one alate and nine immature apterous aphids, taken from wheat plants held in the laboratory, were released onto each plant and cages were put in place as described above. One week later, a further five aphids were released onto each plant. The covers were removed on 29 November and the number of *R. padi* and a natural infestation of *M. dirhodum* were counted. The number of tillers per plant was also recorded.

The second experiment was set up in late December using the same plants as in the first experiment. Prior to the start of the experiment, the plants were lightly trimmed to 8–10 cm in height and any *M. dirhodum* present were removed. The plants were transferred to a controlled environment room, at 18 °C with a 14:10 light: dark cycle and were placed in replicate rows on a metal tray. One mature, apterous nymph or adult *R. padi*, 2 mature, alate nymphs and one alate adult were released on each plant. The aphids had been removed from cereal plants in a glasshouse on 11–12 December and kept on damp filter paper in Petri dishes in the 20 °C CE room to eliminate the parasitized individuals. The plants were not covered and were bottom watered as required. After 4 weeks, all aphids were suctioned from each plant, collected into 70% ethanol, and retained for a later counting. The tillers were counted, then two tillers per plant were harvested and immunoblotted to check for the presence of endophytes.

Data on the effects of loline-producing endophytes in *Elymus* spp. and *S. cereale* on aphids and spittlebugs were checked graphically for normality and homogeneity and then analyzed with a one-way analysis of variance in Genstat V21. The means were separated using Fisher’s post hoc test. Data for the second rye experiments were log-transformed (ln+1) to stabilize the variance and then analyzed using Restricted Maximum Likelihood (REML) in Genstast V16, with Endophyte as fixed effects and Replicate and Sampling Date as random effects.

### 2.3. Uptake of Lolines by Grasses and Cereals

In this experiment, the seed of rye (cv Rahu), tall fescue (cv Jesup), and ryegrass (cv Grasslands Samson) with and without endophyte infection were germinated on damp filter paper in Petri dishes and then planted into a seed-raising mix (Daltons™) in 7 cm diam. Petri dishes in spring (early September) 2015. The plants were immunoblotted on 28 October and 4 November. Wheat (cv Monad) not infected with endophyte was also included. The Petri dishes were upright with a hole in the side of the lid and base through which the tillers grew. There were 14 replicates for each of the 14 treatments, with treatments randomized within 14 replicate rows. The plants were watered regularly with 2 mL of water delivered into each Petri dish with an auto pipette.

A pre-treatment count of a natural infestation of aphids (predominantly *R. padi*) on each plant was made in situ on 14 October 2015 when the plants were 4 weeks old. Two days later, the first application of a solution containing 1000 µg g^−1^ of loline was made to half of the plants for each treatment. The solution was prepared using pure lolines that had been stored at 4 °C. Distilled water (100 mL) was added to a bottle, followed by 2 × 100 µL of loline solution and then another 100 mL of water. The bottle was swirled gently to mix. A total of 2 mL of loline solution was delivered through to the potting medium for each treated plant with an autopipette. After the lolines were added, a clean pipette tip was used to deliver 2 mL of water to each untreated plant in the same way. The lolines were applied on 16 and 18 October and the aphids were counted again 2 days after the second application.

For the loline application experiment, all statistical computations and data handling operations were performed using R version 4.2.0 [16]. A generalized linear model (GLM) was used to analyze the total number of aphids counted in situ per tiller, including the senescent leaves. The numbers of aphids per tiller pre- and post-application of the lolines were analyzed using the GLMs estimated by the maximum likelihood (ML) with a negative binomial distribution and log link function. Each model contained ‘Plant species—endophyte’ (factor with seven levels: ‘Rahu E−’, ‘Rahu E+’, ‘Ryegrass E−’, ‘Ryegrass E+’, ‘Tall fescue E−’, ‘Tall fescue E+’, and ‘wheat’), and ‘Column-block’ (i.e., a randomized complete column-block factorial) as the explanatory variables. The log of the number of tillers was included as an offset variable since the number of tillers differed per observation. An over-dispersion was detected, and a negative binomial GLM was applied. Since we had two replicates of each treatment level (‘Plant species—endophyte’) within a column-block, the five response variables were averaged in each column-block using plant species–endophyte and then a negative binomial GLM was fitted using the R package MASS [17]. For the analysis of the number of aphids post-application of the lolines, the factor ‘loline application’, with two levels ‘Nil’ and ‘Plus’ and their interaction, were added as the explanatory variables. The log of the number of tillers was included as an offset variable as the number of tillers differed per observation. An over-dispersion was again detected, and a negative binomial GLM was applied.

For all models, graphical model validation tools such as plots of residuals against the fitted values, standardized residuals versus the fitted values, quantile–quantile plots, residuals against leverage, and residual diagnostics based on a simulation-based approach [R package DHARMa [18]], were used for the model’s validation. In all GLMs, the significance of the fixed terms was assessed via a backward variable selection using a likelihood-ratio test and Akaike’s Information Criterion (AIC) [19], and the significant terms were followed up by applying a multiple-comparison procedure using Tukey adjusted contrast [20].

For a significant two-way interaction term for the analysis of the number of aphids after the application of the loline alkaloids, the multiple comparison was conducted in two ways, allowing for comparisons between (i) the loline application levels within the plant species–endophyte (i.e., the comparisons of the levels of loline were conducted separately for each level of plant species–endophyte, e.g., ‘Nil’ versus ‘Plus’ within ‘Rye E−’ level), and (ii) between the plant species–endophyte levels within the loline application levels (i.e., the comparisons of the plant species–endophyte were conducted separately for each level of the loline application, e.g., ‘Rye E−’ versus ‘Rye E+’ versus ‘Ryegrass E−’ versus ‘Ryegrass E+’ versus ‘Tall fescue E−’ versus ‘Tall fescue E+’ versus ‘Wheat’ within ‘Plus’ the loline application level).

## 3. Results

### 3.1. Loline-Producing Epichloë Endophytes in Wild Elymus spp. and Rye

There were no significant differences in the numbers of *R. padi* on plants with and without endophyte for each of the different *Elymus* species/endophyte combinations (*p* > 0.05) (Table 1). The mean loline alkaloid content of the whole tillers varied from 299 µg g^−1^ in *E. caninus* to >600 µg g^−1^ in two of the *E. mutabilis* accessions (Table 1).

**Table 1:** Mean log transformed (Ln + 1) numbers of *Rhopalosiphum padi* aphids per plant (±standard error of the mean) (SEM) in different *Elymus* species with and without endophyte and concentration of loline alkaloids in whole tillers from the different accessions.

Species	Endophyte	Mean No (Ln + 1) *R. padi* Plant^−1^						Total Loline (µg g^−1^)
		E+ ± SEM		N ^1^	E− ± SEM		N ^1^	E+
*E. mutabilis*	AR3048	3.85	0.67	5	3.09	1.47	1	683
*E. mutabilis*	AR3050	1.68	0.44	12	2.28	0.86	3	642
*E. mutabilis*	AR3068	2.75	1.04	2		*	0	305
*E. uralensis*	AR3067	2.37	0.42	14	1.40	0.66	4	380
*E. caninus*	AR3074	3.57	0.44	13	*	*	0	299
*E. mutabilis* var. *oschensis*	AR3076	3.45	0.44	12	*	*	0	357

* 0 plants available ^1^ No. of plants per treatment.

In the first rye experiment, all aphids were confirmed to be *R. padi*. The number of aphids per plant and per tiller did not differ significantly between the rye infected with endophyte AR3046 and that which was endophyte-free (Table 2). The lack of an effect on the aphids was also found in the second experiment in which eight different accessions were screened for their effects on the aphids. The number of aphids did not differ between the accessions or due to the endophyte status (data not presented).

### 3.2. Comparative Effects of Endophyte on Aphids and Spittlebug

The mean scores for the amount of spittle produced (Globule score) on Day 1 and Day 2 was significantly less on the rye infected with AR3046 than on the endophyte-free rye for the first two assessments, whereas there was no difference between the endophyte-infected and endophyte-free meadow fescue (Table 3). A further analysis found no significant difference in the number of globules that were small or medium-sized for spittlebugs which were fed endophyte-free rye compared with those fed endophyte-infected rye. There was a significant reduction, however, in the number of large globules produced by spittlebugs which were fed endophyte-infected rye compared with those fed endophyte-free, but no difference was detected for the meadow fescue. The day after the experiment was set up, the total number of spittlebug nymphs and adults was lower on the rye infected with AR3046 than those on endophyte-free plants, but there was no significant difference in the numbers on the endophyte-infected and endophyte-free meadow fescue. The proportion of the total number that were adults was also significantly lower on those fed endophyte-infected rye. By day 4, most of the spittlebugs had developed into adults and had ceased to feed.

### 3.3. Uptake of Applied Lolines

**Figure 1:**
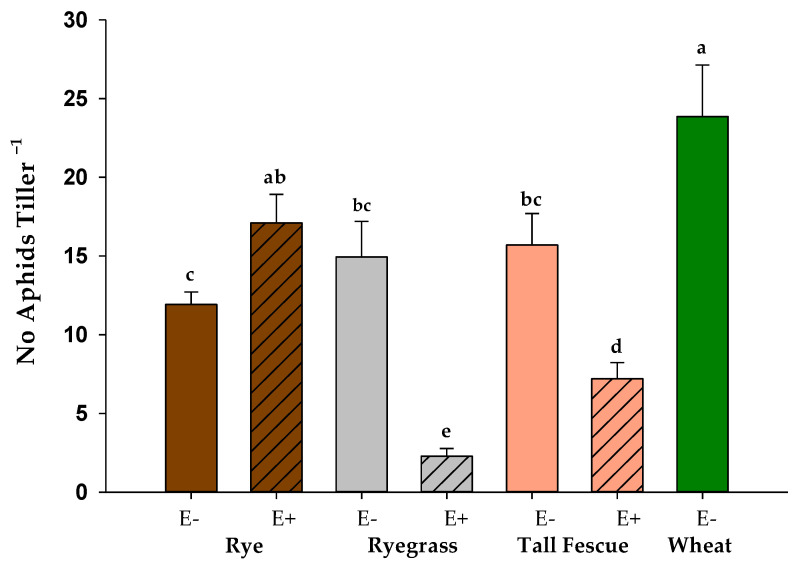
Mean number of *Rhopalosiphum padi* per tiller ± standard errors pre-application of lolines to rye, ryegrass, tall fescue (with (E+) and without endophyte (E−)), and endophyte-free wheat. Different lowercase letters indicate statistically significant differences between plant species and endophyte (multiple comparison procedures were performed using Tukey adjusted contrast at α = 0.05).

The plant species and endophytes had a highly significant effect on the total number of aphids per tiller (*p* < 0.001) pre-application of lolines. For both ryegrass and tall fescue, the number of aphids per tiller was significantly lower on endophyte-infected plants compared to those that were endophyte-free (*p* < 0.05; Figure 1(b,c)). In contrast to this, there were significantly fewer *R. padi* on the endophyte-free rye than on endophyte-infected rye (*p* < 0.05; Figure 1).

**Figure 2:**
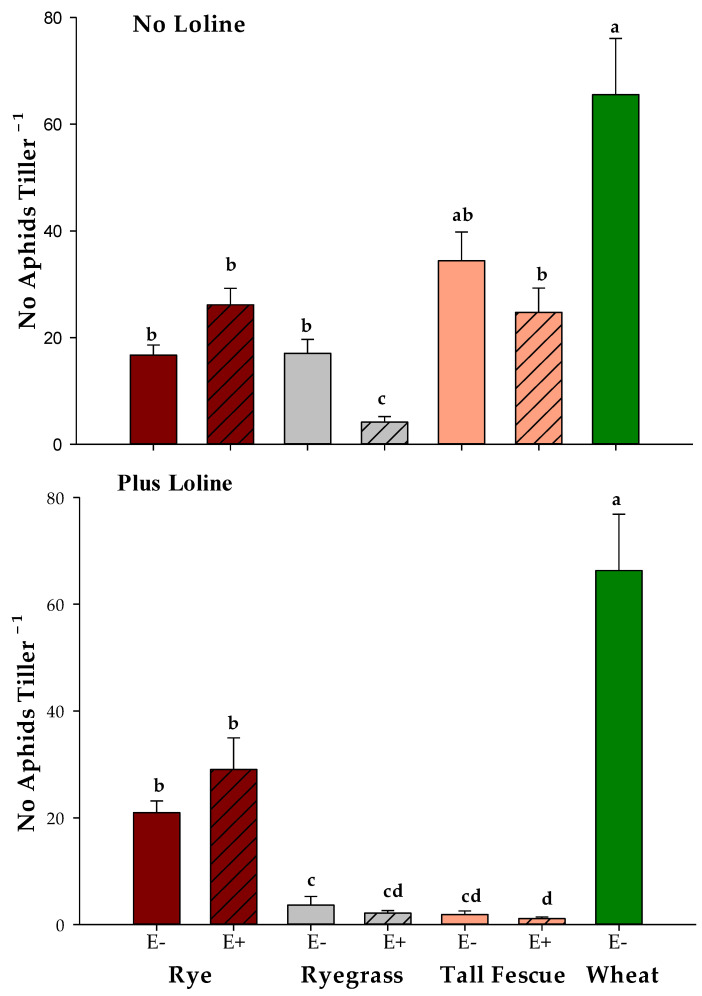
Mean number of *Rhopalosiphum padi* per tiller ± standard errors post-application of lolines to rye, ryegrass, tall fescue (with (E+) and without endophyte (E−)), and endophyte-free wheat compared with controls that did not receive lolines. Different lowercase letters indicate statistically significant differences between plant species and endophyte levels within loline application (multiple comparison procedures were performed using Tukey adjusted contrast at α = 0.05).

After the lolines’ application, there were highly significant interactions between the plant species, endophyte, and loline application for the number of aphids per tiller (*p* < 0.001; Figure 2). The number of aphids on both the endophyte-free ryegrass and tall fescue were reduced by the lolines to the extent that the aphid numbers were significantly less than their loline-free counterparts, but this did not reduce the numbers on the rye irrespective of the endophyte status (Figure 2). Similarly, the application of loline to wheat had no significant effect on the *R. padi* numbers. Of interest, however, was the observation that the number of *R. padi* per tiller was significantly higher on wheat than on rye, suggesting that rye was a less favorable host.

## 4. Discussion

Our research has unexpectedly shown that the lolines produced by endophytes in *Elymus* spp., a natural Triticeae host, has had no effect on the aphid *R. padi,* despite the lolines they produced being at bioactive concentrations [8]. Furthermore, when these endophytes were inoculated into rye, there was no effect on the aphid populations, whereas xylem-feeding spittlebugs fed less, and their numbers were reduced compared with the Nil endophyte controls. Conversely, meadow fescue infected with a loline-producing endophyte affected the aphids but not the spittlebug. We also applied loline alkaloids to the surface of the potting medium of young ryegrass, tall fescue, and rye, and conducted pre-and post-application counts on the number of *R. padi* aphids present on the plants. The lolines taken up by the plants reduced the aphid numbers on the endophyte-free ryegrass and tall fescue to levels similar to those on the endophyte-infected plants without lolines but failed to have any effect on the aphid numbers on rye plants. Matsukura et al. [21] in experiments in which four vascular-feeding Clypeorrhynchan pests were fed Italian ryegrass (*Lolium multiflorum*) infected with *E. uncinata*, showed significant decreases in the survival rate in three of the species. Two of the affected insect species were known to be phloem-feeders and the other was suspected to be a phloem feeder due to a high sugar content in its wastes. The one species that was not affected by the Italian ryegrass infected with loline-producing *E. uncinata* was known to prefer to feed in the xylem. The survival of all four species, however, was affected by lolines when they were fed to the insects in parafilm sachets, showing that a lack of sensitivity to lolines was not a consideration.

There is very little information in the literature on the effects of endophyte in *Elymus* on insects, although it is known that wild grasses such as *Elymus* commonly harbor BYDV [22], thus providing a source of infection for susceptible hosts, including wheat and barley. One survey showed that 100% of the seeds collected from Canadian wild rye (*E. canadensis*) across seven different states in the USA contained endophyte [23]. Such high infection levels are usually associated with advantages that endophytes confer on their hosts. Curiously, aphids were rare amongst the plants surveyed in this study with those at the only site where aphids were found thought to be ‘non-colonizing’ aphids that were feeding temporarily on the plants but that were still capable of transmitting the virus. We conclude from this that, despite rye and *Elymus* being within the same tribe and collectively within the same sub-family as Poeae, there has been little selection pressure to develop a resistance to aphids via an endophyte infection.

A similar large-scale screening of wild meadow fescue collections in seven European countries had average endophyte infection rates between 69% and 91%, with a small but significant correlation between the genetic diversity and geographic location [24]. The meadow fescue endophyte AR1006 used in the experiments reported here is exclusively vertically transmitted and produces high concentrations of loline alkaloids [15] similar to the high concentrations reported by Cagnano et al. [24]. Meadow fescue infected with endophytes such as this are resistant to aphids and many other insects [6].

It is generally accepted that phloem sap flows from the leaves to other organs, including the roots, whereas the xylem is drawn up from the roots to the upper parts of the plant. In this context, it is interesting that the lolines applied to the plant roots were still partitioned differently between the grasses and rye and in the same way as when they were produced by the endophyte. The endophyte had no influence on this process as the uptake and distribution of the lolines was the same in the endophyte-infected and endophyte-free plants.

The growth of *Epichloë* endophytes within their hosts is confined to the intercellular spaces with hyphae attached to the cell walls and the growth of the endophyte synchronized with that of its host [25]. They do not usually colonize the vascular system, but the alkaloids they produce can be translocated within their host via the vascular system. Based on their activity against phloem-feeding aphids and xylem-feeding spittlebug, it is apparent that for the three genera investigated in the Triticeae, the lolines were most likely present in the xylem, whereas for those in Poeae the lolines were, as expected, in the phloem. Nevertheless, we do not know why the translocation of the lolines at least in *Elymus* is preferentially in the xylem where it is less accessible to aphids and similarly in an artificially created symbiosis with rye. For these species, this has implications for the use of endophytes to control aphids which not only have damaging effects on the plants through their feeding, but more so due to the transmission of viruses such as BYDV [26]. The absence of any effect on aphids from the uptake of lolines in wheat suggests that this species acts in a very similar way to *Elymus* and rye. Other genera within the Triticeae that are known to harbor endophytes include *Hordeum, Agropyron, Elytrigia*, and *Triticum* [27]. Clement et al. [28] found the aphid densities to be lower on endophyte-infected *H. bogdanii* and *H. brevisubulatum* subsp. *violaceum* compared to their endophyte-free conspecifics. Prior to this research, N-formylloline had been isolated from both endophyte-infected *H. bogdanii* and *H. brevisubulatum* subsp *violaceum* [29] and although these endophytes also produced ergovaline, the lolines were considered to be the most likely to have had the antibiosis effect observed with *R. padi* [28]. The further testing of *Epichloë*-infected wild barley lines has provided mixed results for the effects of aphids, but their status regarding the production of lolines is unknown [30,31]. The results of the current study indicate that the hosts in the Triticeae tribe partition lolines into the xylem, though this has not been investigated for *Hordeum*. Furthermore, this needs to be confirmed by measuring the loline content of the phloem and xylem in different species. Clearly, further research is needed to determine whether the loline partitioning into xylem or phloem is consistent in Poaceae and Triticeae tribes, respectively.

## Figures and Tables

**Table 2:** Number of rye plants infected with *Epichloë mutabilis* endophyte AR3046, number inoculated but not infected (E−) and number not inoculated (Nil), number of tillers per plant, and number of aphids per plant and per tiller.

Treatment	No. Plants	No. Tillers Plant^−1^	No. Aphids Plant^−1^	No. Aphids Tiller^−1^
AR3046	12	7.4	47.3	6.9
E− ^1^	12	9.9	33.9	4.9
Nil ^2^	5	8.8	38.2	3.9
Max SED		2.59	18.93	2.70
*p*		NS	NS	NS

^1^ Plants inoculated with AR3046 that failed to become infected. ^2^ Uninoculated rye plants.

**Table 3:** Impact of lolines in rye produced by *Epichloë mutabilis* and meadow fescue produced by *E. uncinata* (E+) compared with endophyte-free (E−) counterparts on spittlebug feeding, development, and survival.

Treatments	No Globules			Globule Score		Spittlebug	
	Small	Medium	Large	Day 1	Day 2	No.	Propn Adults
Rye E+	1.83	2.83	0.33	1.67	1.83	1.17	0
Rye E−	1.67	2.33	3.33	7.17	4.17	2.83	0.36
Meadow fescue E+	3.17	0.67	0.67	2.83	1.83	2.83	0.17
Meadow fescue E−	2.17	0.83	0.67	4.17	1.50	2.50	0.44
SED	1.009	0.882	0.758	0.845	0.909	0.322	0.14
*p* (<0.05)	NS	NS	0.004	<0.001	0.036	<0.001	0.023

## Data Availability

Two sets of data that showed no differences between treatments have not been reported in the paper but can be obtained through a request to the corresponding author.
